# Translating advances in microbial bioproduction to sustainable biotechnology

**DOI:** 10.3389/fbioe.2022.968437

**Published:** 2022-08-23

**Authors:** David N. Carruthers, Taek Soon Lee

**Affiliations:** ^1^ Joint BioEnergy Institute, Emeryville, CA, United States; ^2^ Biological Systems and Engineering Division, Lawrence Berkeley National Laboratory, Berkeley, CA, United States

**Keywords:** synthetic biology, bioproduction, life cycle assessment (LCA), techno-economic assessment (TEA), bioeconomy

## Abstract

Advances in synthetic biology have radically changed our ability to rewire microorganisms and significantly improved the scalable production of a vast array of drop-in biopolymers and biofuels. The success of a drop-in bioproduct is contingent on market competition with petrochemical analogues and weighted upon relative economic and environmental metrics. While the quantification of comparative trade-offs is critical for accurate process-level decision making, the translation of industrial ecology to synthetic biology is often ambiguous and assessment accuracy has proven challenging. In this review, we explore strategies for evaluating industrial biotechnology through life cycle and techno-economic assessment, then contextualize how recent developments in synthetic biology have improved process viability by expanding feedstock availability and the productivity of microbes. By juxtaposing biological and industrial constraints, we highlight major obstacles between the disparate disciplines that hinder accurate process evaluation. The convergence of these disciplines is crucial in shifting towards carbon neutrality and a circular bioeconomy.

## Introduction

Industrial decarbonization is crucial for combating anthropogenic climate change. Recent assessments published by the Intergovernmental Panel on Climate Change have inspired international pledges for global carbon emissions reduction that generally aim to mitigate global warming to 1.5°C as per the Paris Accords (Masson-Delmotte et al., 2018; Hoegh-Guldberg et al., 2019). Despite near universal recognition of the deleterious effects of climate change, pledges for emissions reduction, and ratification of the UN Sustainable Development Goals, actualizing climate commitments has proven extraordinarily challenging in part due to the global inertia of a fossil-based economy (Peters et al., 2019). Today, approximately 90% of chemicals are generated from fossil fuels as carbon and energy feedstocks (Blank et al., 2020).

Recent proposals have advocated shifting material flows towards a circular economy through upcycling of waste streams, increasing efficiency, and shifting raw material acquisition to biobased pathways. A major advantage of bioproducts is the ease with which they may be substituted into traditional petrochemical systems as “drop-in” alternatives. Drop-in bioproducts have minimal impact on infrastructure, contemporary behavior, or sociopolitical attitudes and often maintain lower carbon emissions. The substitution of petrochemicals with biobased alternatives could reduce fuel associated greenhouse gas (GHG) emissions by 75–80% (Balakrishnan et al., 2015) and further substitution of bio-based plastics could reduce plastic production GHG emissions by 66% (Spierling et al., 2018; Zheng and Suh, 2019).

Metabolic engineering is a promising strategy for the delineation and decarbonization of plastics and fuels by supplanting high volume chemical production with biosynthetic routes. The last decade has witnessed a veritable revolution in synthetic biology, characterized by the relative ease with which microbes can be modified and optimized for high titer bioproduction via the canonical design-build-test-learn (DBTL) cycle, utterly transforming our ability to engineer complex biosynthetic pathways (Cameron et al., 2014; Robinson et al., 2020). Perhaps most remarkable is the horizontal expansion of metabolic pathways to generate a diverse portfolio of chemicals with the propensity to disrupt the traditional manufacture of food, fuels, materials, and medicines (Voigt, 2020). Recent synthetic biology advances like genome editing [CRISPR(Sander and Joung, 2014) (Kim J. et al., 2021); CRISPRi (Wu et al., 2017) (Banerjee et al., 2020)], adaptive laboratory evolution (Mohamed et al., 2020), the explosion of computationally informed protein folding prediction software (Jumper et al., 2021), and advanced library screening technologies (Saleski et al., 2021) have cumulatively improved sustainable bioproduction platforms. Specifically, metabolically engineered microbes have demonstrated generation of food supplements (Torres-Tiji et al., 2020), biofuels for blendstocks [e.g., limonene (Alonso-Gutierrez et al., 2013), isoprenol (Kim J. et al., 2021), isobutanol (Saleski et al., 2021)], biopolymers [e.g., PHA (Aurisano et al., 2021)], and bulk chemical precursors [e.g., adipic acid, lactic acid, l-lysine 3-hydroxypropionic acid, among others (Bozell and Petersen, 2010)]. This array of bioproducts is complemented by diverse engineering strategies. The most common approach is a consolidated rewiring of a microbial chassis for enhanced bioproduction, though researchers have also pursued the integration of variable bioproduction routes within the same chassis (Lee and Kim, 2015) as well as overhauling carbon assimilation to diversify substrate availability (Kim et al., 2020). At a community level, investigations of the rhizosphere have helped to identify constitutive microbes, nutrient exchanges, and microbial interactions that may improve community-level production, stability, and nutrient recycling thereby reducing material inputs (Ahkami et al., 2017; Lawson et al., 2019).

Despite significant improvements in pathway engineering, few platforms have proven commercially viable due in part to the lack of accessible economic assessments that address early bottlenecks and adequately scale laboratory cultures to pilot settings (Lynch, 2021). This deficiency is coupled with the difficult task of actually determining whether a bioproduction platform is more sustainable than current practices, which historically is not necessarily the case (Searchinger et al., 2008; DeCicco et al., 2016). Thorough accounting requires not only the quantification of cost, energy, and emissions throughout the process, but the inclusion of nontrivial yet ambiguous inputs like, for example, land-use-change, which considers how a production platform displaces native habitat. And, often most importantly, the purported sustainability of a process must align also with its commercial viability. Summarily, emerging bioproduction platforms are confronted with the formidable task of simultaneously approaching carbon neutrality while maintaining price parity with long established chemical syntheses (Lee et al., 2021).

Life cycle assessment (LCA) and techno-economic assessment (TEA) are the two dominant strategies for quantifying the relative environmental and economic costs of a production strategy. While TEAs have been methodologically applied throughout the industrial era, LCAs are relatively new and have matured from a Boolean choice between products to a multifaceted environmental report. In this review, we first describe how LCAs and TEAs are traditionally conducted to yield empirical metrics and their extrapolation to synthetic biology with emphasis on biofuels, biopolymers, and biochemical precursors of specific importance. We further elaborate on recent metabolic engineering of microorganisms for enhanced bioproduction, namely through the improvement of titer, rate, yield, and substrate utilization with specific attention to studies conducting LCAs/TEAs. Finally, we underline the necessity of assessment harmonization, accessibility, and transparency to better translate results between disciplines and, quintessentially, facilitate proper allocation of research investment for informed policy decision making.

## Economic and environmental evaluation of bioproduction platforms

Both LCA and TEA aim to provide practitioners with useful indicators for improving the affordability or sustainability of a given process. Importantly, assessment accuracy is contingent on data reliability and availability, factors that can prove inhibitive for bioproduction platforms at low technology readiness levels (TRLs) (Guinée et al., 2011; Thomassen et al., 2019). Similarly, the integration of LCA and TEA has also been recognized as a major obstacle in assessing the combined economic and environmental burdens of specific processes (Mahmud et al., 2021). Here, we provide an overview of the traditional LCA, TEA, and then explore how recent assessments have addressed the challenges of emerging bioproduction technologies.

### Life cycle assessment

The nominal strategy for determining bioproduct sustainability is through an LCA, a technique that originally arose as a tool for product selection based on potential environmental impact (e.g., “paper or plastic?”) (Guinée et al., 2011). Though the philosophy behind LCA has matured over the last several decades, the overall framework remains the same. A product life cycle consists of five key stages: raw material acquisition, manufacturing, packaging and transportation, use-phase, and end-of-life. The LCA framework is qualitatively outlined by ISO (International Standards Organization) 14040:2006 and 14044:2006 with intentional ambiguity to enable its translation to various production pathways, inputs, impacts, and general assumptions (Standardization International Organization for, 2006). At its core, the framework is remarkably simple and consists of four principal components:

1. Scope and Goal: A system boundary and functional unit of production are allocated as a basis for process calculations. The system boundary dictates the overall processes to be considered and may extend from raw material acquisition to the final product (“cradle-to-gate” or “well-to-pump” for fuels) or to the use-phase and end-of-life (“cradle-to-grave” or “well-to-wheels” for fuels).
2. Life Cycle Inventory (LCI): The inventory accounts for the material and energy flows within the system boundary. Several LCI software packages have been developed to facilitate practitioner accounting and are especially valuable for multi-input multi-output mass and energy flows (Suh and Huppes, 2005). Many dedicated LCI packages have been developed, including the popular EcoInvent v3.1 (Wernet et al., 2016), which is used for commercial product generation. However, practitioners often choose more robust process engineering tools like Aspen Plus^®^ and SuperPro Designer^®^ that account for mass and energy flows with thermodynamic modeling.
3. Life Cycle Impact Assessment (LCIA): Next, specific impacts of interest are selected to measure the effects of the functional unit within the system by coupling environmental allocations to materials and energy sources. The impacts usually include specific, physical midpoint indicators like greenhouse gas emissions (CO_2_eq), water intensity, and energy return, though may extend to environmental factors such as eutrophication potential, human toxicity, acidification, and direct or indirect land use change. LCIAs may also include more ambiguous endpoint indicators such as biodiversity loss, ecosystem damage, and human health effects through defined weighting of midpoint indicators (Souza et al., 2015). Common impact databases include Tool for Reduction and Assessment of Chemical and other environmental Impacts (TRACI) by the US EPA (Bare, 2011) and ReCiPe 2016 (Huijbregts et al., 2017) among others. Traditionally, bioproduction assessments utilize midpoint indicators to communicate the relative change in emissions and energy return between production systems. More recently, works have employed neural networks to better understand how different inventories characterize chemicals and thus predict impacts of novel chemical syntheses (Song et al., 2017).
4. Interpretation: Lastly, comparative impacts are weighed within the context of the system inventory and boundary to inform process selection, policy, and investment decisions (e.g., does the bioproduct have lower carbon intensity compared to its petrochemical analogue?).

The life cycle framework may also be subdivided into either an attributional LCA (aLCA) that allocates the environmental impacts of a production pathway to a given project or a consequential LCA (cLCA) that considers how environmental impacts change in response to product generation, the energy/material displaced by the product, and product demand (Earles and Halog, 2011). Each category has distinct importance for process interpretation and intercommunication between the four individual components is crucial for improving confidence in LCA results.

Several overarching software tools have been developed for LCA, including openLCA (https://openlca.org/) and SimaPro (https://simapro.com/). Such tools facilitate inventory construction and selection of impact indicators from a library of different methodologies. In the case of bioproducts, Greenhouse gasses, Regulated Emissions, and Energy in Transportation (Greet, 2022), a tool published by Argonne National Laboratory, is popularly applied to draft emerging bioproduction schemes including biofuels ranging from corn ethanol as a gasoline additive to algal biofuels for biodiesel (Argonne GREET Model). It has also been employed in conjunction with Aspen Plus^®^ to evaluate bioproduction of 12 high performance platform chemicals (Dunn et al., 2015).

In general, the flexibility of ISO 14040 complements the diversity of bioproduction platforms, which generate not only disparate products, but utilize dramatically different feedstocks from climatically distinctive regions (e.g., Brazilian sugarcane vs. American corn). Practitioners therefore rely upon sensitivity analyses like Monte Carlo simulation and scenarios forecasting to elucidate inputs that disproportionately affect impacts (Patouillard et al., 2019). For LCA, scenarios may present different conversion rates, throughput, recycling fraction, or total yields (Krömer et al., 2020). Increasing emphasis has been placed on the inclusion of LCA in academic publications as an important tool for guiding policy as well as further research and development (Subramaniam et al., 2021). Nonetheless, accurate environmental accounting of diverse and numerous bioproduction platforms represents a daunting challenge.

### Techno-economic assessment

While LCAs are crucial for comparing the relative sustainability of biological and petrochemical products, TEAs actualize the commercial viability of biotechnology platforms. TEAs differ from other accounting strategies (e.g., cost-benefit analysis or cost-effectiveness analysis) by focusing on emerging technologies and, in general, there are few explicit methodological guidelines (Van Dael et al., 2015). TEAs tend to be highly specialized to less mature production pathways and are typically completed at lower TRL to assess early design decisions (Scown et al., 2021). Depending on TRL, TEA may be used for attracting stakeholders and encouraging investment by governmental entities, research and development departments, or early-stage investors to ensure efficient technology maturation (Sick et al., 2020).

At their most basic level, TEAs help to identify economic indicators in production pathways to reduce the minimum selling price (MSP) of a given product. The MSP is the price at which the net present value of a production system is zero and, when compared to market prices, the MSP succinctly describes the profitability of that system. While TEAs parallel the same overall design logic as LCAs in their accounting of material and energy flows, they maintain obvious emphasis on reducing overall process expenditures rather than environmental burden. Unlike the clear methodological demarcation of LCAs, TEA methodologies are far less uniform, often owing to divergent objectives depending on TRL (Thomassen et al., 2019). Scenarios forecasting, for example, is often contingent upon market uncertainty, which is especially relevant for bioproducts competing with volatile petrochemical prices prone to significant fluctuations in global supply and demand. Furthermore, price modeling must account for coproducts (Biddy et al., 2016).

Techno-economic assessments divide costs into two categories: capital expenditures (CAPEX) that include fixed assets and operational expenditures (OPEX) that encompass feedstock prices, utilities, operator salaries, and similar day-to-day expenses (Lynch, 2021). CAPEX may be further expanded by estimating asset interest rates and modified accelerated cost recovery system (MACRS) for system cost depreciation. Calculation of total expenditures within a discounted cash flow analysis based on an internal rate of return enables the elucidation of the MSP of a product at scale in an “nth” plant which is readily comparable to current price of the good in the market. Green technologies range from carbon capture to lithium-ion batteries to lignocellulosic biofuels, all of which are geared at displacing current technologies for emissions reduction. TEAs are therefore a pivotal tool in separating viable production by quantifying the current state of technology and highlighting design areas for the most efficient improvement. Recently, TEA methodologies have been tutorialized for practitioners to better assess obstacles and opportunities in low TRL bioproduction (Buchner et al., 2018; Thomassen et al., 2019).

### Translation of life cycle assessments and techno-economic assessments to biological systems

Initial considerations of biochemical sustainability date to the development of the 12 principles of green chemistry (Anastas and Williamson, 1996), which were gradually parametrized in early LCA frameworks and ultimately applied to industrial ecology as a whole (Anastas and Lankey, 2000). Microbial metabolic engineering has the propensity to generate economically competitive products with vastly improved sustainability metrics compared to archetypical petrochemical production (Dunn, 2019). Assessments of biological systems traditionally focus on agricultural feedstocks and biofuels, but more recently have expanded to a broader range of bulk, value-added chemical precursors (Dunn et al., 2015).

The central challenge to integrating synthetic biology within the TEA/LCA framework is the mitigation of uncertainty. Longstanding challenges for synthetic biologists, for example, have been the unpredictability, incompatibility, volatility, and lack of knowledge in complex circuits that complicate the extrapolation of bench to pilot scale production (Kwok, 2010) as well as experimental reproducibility (Baker, 2016). Physical constraints also strongly influence bioproduction at scale. Large-scale bioreactors can exacerbate expression stochasticity due to gradients in dissolved gas and nutrient concentrations, which lend to suboptimal production and potentially the selection of deleterious mutations (Wehrs et al., 2019; Czajka et al., 2020). This phenomenon is especially well-studied in *E. coli*, where large-scale production often results in lower overall biomass and growth rates accompanied by significant organic acid accumulation (Neubauer et al., 1995).

From an analytical standpoint, the difficulty of intercomparing assessments cannot be understated. Even when utilizing the same production pathway and operating in accordance with ISO 14040:2006, the extrapolation of low confidence assumptions from low TRL, bench scale production strategies often yields assessments with starkly different conclusions and suboptimal design decisions (Hellweg and Milà i Canals, 2014).

Algal biofuels remain a useful case study for effective translation of LCA/TEA to biological systems. Algal biofuels continue to be popular candidates for LCA due to the abundance of extraction methods (e.g., hydrothermal liquefaction, lipid extraction, or combined algal processing), strains (e.g., halophilic *Dunelia* sp. or freshwater *Chlorella* sp.), and culture conditions (e.g., outdoor pond, outdoor raceway, photobioreactor) alongside the ease with which algal biodiesel can be integrated into contemporary fuel systems for valorization of photosynthetically sequestered CO_2_. However, meta-analyses and harmonization reports have highlighted how the range of modeling assumptions, process data, and altogether divergent production schemes can heavily impact the translation of experimental data from flask to field (Quinn and Davis, 2015; Tu et al., 2017; Cruce et al., 2021). Quinn et al. note the variability in TEA/LCA metrics, citing reports of GHG emissions varying from -75–532 g CO_2_eq per 1 MJ fuel as well as approximately $1.34 to $30 per gallon of fuel (Quinn and Davis, 2015).

More broadly, assessment variability is ubiquitous across bioproducts. A recent bioplastic critical review further reported high variation between production strategies with energy usage per kilogram of bio-PET varying by 400% and bio-PLA by over 1,000% (Walker and Rothman, 2020). Harmonization reports have attempted to synthesize these data to better assess the state of technology and note areas of greatest sensitivity. In the case of algal bioproduction, harmonization reports have emphasized pond productivity as and material inputs as major targets for optimization (Cruce et al., 2021). Perhaps most importantly, is that these studies stress the necessity for data reliability, transparency, availability, and model homogenization such that assumptions between studies can be controlled for and conclusions better validated. Specific studies elaborating on the complexities and deficiencies typical in current LCAs of bioproduction platforms have also been conducted (McKone et al., 2011; McManus et al., 2015; Ögmundarson et al., 2020).

**FIGURE 1:**
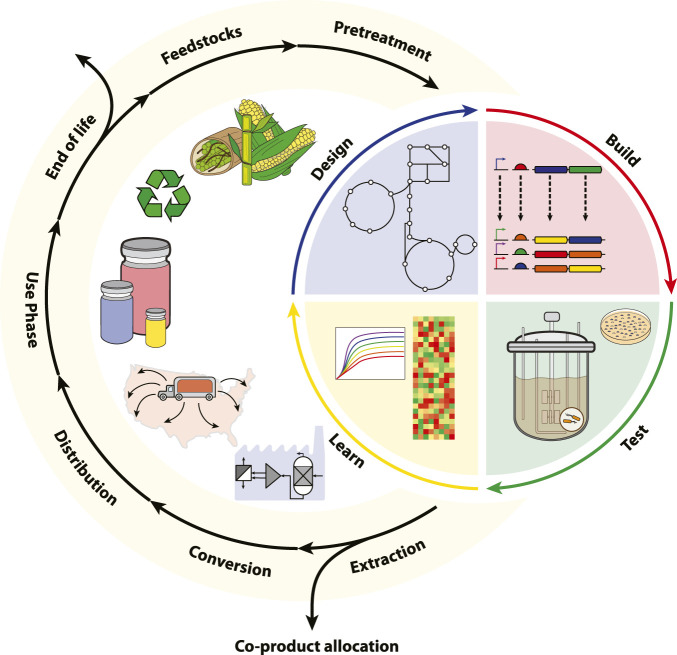
The integration of LCA and synthetic biology within a circular bioeconomy. Here, the LCA framework is subdivided into the cradle-to-grave stages of raw material acquisition (feedstocks and pretreatment), manufacturing (DBTL, extraction, and conversion), distribution, use-phase, and end-of-life. For bioproduction platforms, extraction may be further subdivided into bioseparation and media recycling with co-product allocation also accounting for biological waste treatment. Synthetic biology is represented by the DBTL cycle and integrated into the core manufacturing stage of the LCA. The framework enables iterative benchmarking for scaling bioproduction according toTRL.

Addressing the uncertainty of emergent, low TRL bioproduction platforms is a formidable challenge for TEAs and LCAs, yet early assessments are imperative for spurring investment in the bioeconomy (Hillson et al., 2019). Recent order-of-magnitude type assessments have been developed to better characterize the economic and environmental fingerprint of low TRL biotechnologies (Shi and Guest, 2020; Lynch, 2021; Scown et al., 2021). These “agile TEA-LCAs” aid biologists in addressing process level bottlenecks and viability early in the DBTL pipeline. The integration of synthetic biology and the archetypical LCA are depicted in Figure 1.

Three novel assessment tools of particular importance include the Biorefinery Simulation and Techno-Economic Analysis Modules (BioSTEAM) (Cortes-Peña et al., 2020; Shi and Guest, 2020), the Bioprocess TEA calculator (Lynch, 2021), and Early State Technoeconomic Analysis ESTEA2 (Viswanathan et al., 2020). Their hallmarks include open-source access, good correlations with more robust tools like GREET and Ecoinvent, and tailored design for practitioners grounded in biological sciences. Low TRL bioproduction platforms are typically hampered by high OPEX due to strain performance, which may be characterized by titer, rate, and yield (TRY) per unit feed as well as other process-level functions like nutrient recycling and co-product allocation (Huang et al., 2021). The fact that improving TRY consequentially improves bioproduction environmental and economic metrics is fundamentally intuitive even at bench scale. However, agile assessments are useful in contextualizing how biological improvements translate to changes in process viability early in process design. Using these tools, synthetic biologists can harness the DBTL cycle to enhance TRY and benchmark improvements *in vivo*. This is exemplified by recent application of python-based BioSTEAM to lignocellulosic production of lactic acid and acrylic acid in which practitioners identified that separations efficiency and titer as well as titer and yield to be the greatest opportunities for improvement, respectively (Li et al., 2021). Another study by McClelland et al., consolidated experimental strain engineering and catalyst loading for optimized linear alpha-olefin production, concluding that attaining 40 g/L titer, 0.5 g/L/hr rate, and 80% theoretical yield would enable economic viability (McClelland et al., 2021). Importantly, these assessments provided comparable results to gold-standard tools like ecoinvent and GREET (Bhagwat et al., 2021; Li et al., 2021).

In the following sections we explore how the burden of feedstocks can be addressed through translation of production platforms to inexpensive and prevalent carbon sources, then consider how synthetic biology has improved the TRY of a specific set of bioproducts.

## Advances in feedstock engineering

Costly feedstocks are a major barrier to efficient bioproduction due to agricultural energy accounting (Cherubini et al., 2009). Assessments of sugar substrates for microbial bioproduction are commonly limited to either Brazilian sugarcane or American corn/beet because of their prevalence as feedstocks for bioethanol. These two sources alone have extraordinarily different environmental impacts. Brazilian sugarcane, for example, tends to offset some nonrenewable energy usage (NREU) due to bagasse combustion and its high sugar content, yet quantifying carbon cost is challenging due to land use change (e.g., pasture vs. rainforest), potential biodiversity loss, and so forth (Renouf et al., 2008; Chaplin-Kramer et al., 2017). Corn, on the other hand, has comparatively higher GHGs, NREU, and eutrophication potential due to fertilizer demand but significantly lower water intensity. These disparate environmental fingerprints showcase how sugar feedstocks are complicated by regional differences in sourcing and agricultural practices and, more broadly, market demand and sociopolitical attitudes. Each factor impacts process uncertainty. In any case, pure sugars are an environmentally and economically expensive substrate. The production of raw sugar, namely glucose, routinely accounts for 40–60% of overall bioproduction OPEX as well as considerable NREU and GHG emissions (Tsiropoulos et al., 2013; Gunukula and Anex, 2017; Rios et al., 2021).

Unlocking recalcitrant substrates has tremendous potential for reducing input costs and GHG emissions, valorizing waste streams, and decoupling food from fuel. Yet gaining access to these substrates through hydrolysis or chemical extraction has proven challenging (Jansen and van Gulik, 2014; Lim et al., 2021). In recent years, significant breakthroughs have been made in engineering microbial uptake of atypical carbon substrates in high production chassis as well as integrating production pathways into microbes with atypical carbon pathways. Regardless of approach, the most promising strategies for feedstock cost reduction utilize either lignocellulosic biomass (i.e., second generation biofuel production) or C1 substrates (i.e., CO_2_, CO, CH_4_, methanol, or formate). Furthermore, the engineering of microbial consumption of C1 feedstocks enables conversion of common waste streams to value-added chemicals (Clomburg et al., 2017; Zang et al., 2021).

**FIGURE 2:**
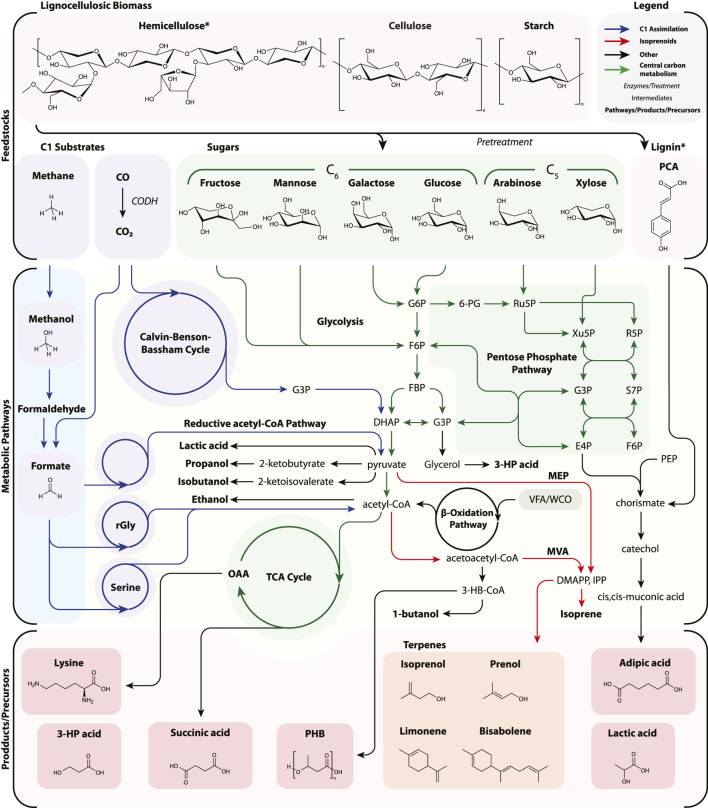
A simplified diagram of feedstock assimilation into bioproduction pathways with specific attention to C1 (CO_2_, CO, CH_4_, methanol, and formate) as well as lignocellulosic biomass. Pathways are not exhaustive nor necessarily the most efficient, but representative to the selection of bioproducts reviewed. Many other routes have been successfully demonstrated. For example, adipic acid production is depicted from *p*-coumaric acid via the shikimate pathway and from lignin derivatives, though may be generated from glucaric acid, TCA intermediates, and the β-oxidation pathway. Lignin is represented by a single aromatic, *p*-coumaric acid, and *cis*,*cis*-muconic acid is usually chemically hydrogenated to adipic acid. Likewise, certain pathways have been simplified for clarity (e.g., lysine and CO assimilation). 3-HP acid, 3-hydroxypropionic acid; 6PG, 6-phosphogluconate; CoA, coenzyme A; CODH, carbon monoxide dehydrogenase; DHAP, dihydroxyacetone phosphate; DMAPP, dimethylallyl diphosphate; E4P, erythrose-4-phosphate; F6P, fructose 6-phosphate; FBP, fructose 1,6-bisphosphatase; G3P, glyceraldehyde-3-phosphate; G6P, glucose-6-phosphate; IPP, isopentenyl diphosphate; MEP, methylerythritol 4-phosphate; MVA, mevalonate; OAA, oxaloacetate; PCA, p-coumaric acid; PEP, phosphoenolpyruvate; PHB, poly-3-hydroxybutyrate; R5P, ribose 5-phosphate; rGly, reductive glycine pathway; Ru5P, ribulose 5-phosphate; S7P, sedoheptulose-7-phosphate; Serine, serine cycle; TCA, tricarboxylic acid cycle; VFA, volatile fatty acids; WCO, waste cooking oil Xu5P, xylulose 5-phosphate.

In this section, we first describe current advances in lignocellulosic bioproduction, then discuss how C1 metabolism could be harnessed to decrease the economic and environmental burden of carbon feedstocks in bioproduction. An overview of the respective feedstocks, metabolic pathways, as well as selected products and precursors is depicted in Figure 2.

### Lignocellulosic biomass

Lignocellulosic biomass is principally composed of cellulose (30–50%), hemicellulose (15–30%), and lignin (15–30%) (Bugg et al., 2011). Cellulose consists of polymeric glucose with 1,4-glycosidic bonds whereas hemicellulose is a heterogeneous, branched polysaccharide comprised of a mix of hexose (e.g., glucose and galactose) and pentose (e.g., xylose and arabinose) sugars. Both lignocellulosic components may be biologically or chemically degraded into utilizable monomeric substrates. Conversely, lignin is an extremely heterogeneous substrate characterized by heavily crosslinked phenolic compounds that contribute to rigidity of woody biomass and have proven especially recalcitrant for microbial production. Collectively, the cost of pretreatment and tolerance remain the principal limitations to the utilization of lignocellulosic biomass for microbial bioproduction.

Deconstruction strategies to catalytically “unlock” lignocellulosic sugar and aromatic monomers may be subdivided into chemical, mechanical, physicochemical, or biological pretreatments with ionic liquids, milling, ammonia fiber explosion (AFEX), or microbial degradation, respectively, as representative examples (Davis et al., 2013; Kumar and Sharma, 2017). Each technology has certain trade-offs, generally pertaining to high cost, efficient conversion strategies as in the use of ionic liquids (ILs), or low cost, slow conversion in the case of microbial degradation (Beckham et al., 2016). Broad LCAs that compare first and second-generation sugar production describe significant variation between processing costs due to choice of deconstruction technology (Bello et al., 2021). Contingent on sourcing, biomass processing results in higher overall GHG emissions and NREU, but comparatively less eutrophication and acidification potential (Bello et al., 2021). Deconstruction reviews have also highlighted the high cost of processing reagents, which are partly due to technology nascency but have spurred optimization studies (Baral et al., 2019). One such study demonstrated efficient deconstruction of sorghum with 50% less IL in a third of the time, then paired the resultant hydrolysate with a naturally tolerant, genetically modified strain of *Rhodosporidium toruloides* (Magurudeniya et al., 2021). The strategy improved bisabolene production on hydrolysate with an estimated 10% lower MSP while further achieving emissions and cost reduction through optimized IL recovery (Magurudeniya et al., 2021). Importantly, integrating deconstruction with bioproduction significantly improved the viability of lignocellulosic production.

While cellulose and hemicellulose are degraded into broadly utilizable hexose and pentose sugars, lignin is degraded predominantly into aromatic compounds like *p*-coumaryl alcohol, coniferyl alcohol, and sinapyl alcohols (Singhvi and Gokhale, 2019). Ideally a strain can simultaneously metabolize hexose and pentose sugars while tolerating concentrations of lignin-derived aromatics that are not only difficult to metabolize but often inhibit microbial fermentation altogether, as is the case for *Saccharomyces cerevisiae* and *Escherichia coli*. Likewise, *E. coli* demonstrates diauxic growth on mixed sugar substrates due to carbon catabolite repression (Deutscher, 2008). Some oleaginous yeasts like *Yarrowia lipolytica* (Sun et al., 2021) and *R. toruloides* (Kirby et al., 2021) have shown high tolerance to lignocellulosic components as well as genetic tractability, with the latter organism having demonstrated terpene production. On the other hand bacterial lignin depolymerization is limited to a subset of α-proteobacteria, γ-proteobacteria, and actinobacteria (Bugg et al., 2011), with *Pseudomonas* sp. As the most popular host. In particular, *Pseudomonas putida* is naturally resistant to ILs due in part to the presence of cholinium catabolizing pathways (Park et al., 2020). Capitalizing on the native predispositions of *P. putida* and *R. toruloides*, adaptive laboratory evolution (ALE) and tolerance ALE (TALE) further improved resistance to ILs (Sundstrom et al., 2018; Lim et al., 2020) as well as enabled growth on the lignin aromatics *p*-coumaric acid and ferulic acid (Mohamed et al., 2020; Liu Z. et al., 2021). In *P. putida*, integration of *xylAB* genes for xylose catabolism enabled simultaneous uptake of hexose, pentose, and aromatic compounds from hydrolysate (Elmore et al., 2020). Simultaneously, other works have expanded the repertoire of *P. putida* and *R. toruloides* bioproducts with value-added muconic acid and terpenoids (Sundstrom et al., 2018; Johnson et al., 2019; Kirby et al., 2021).

Although TALE has increased IL tolerance in *E. coli*, uptake of recalcitrant aromatics remains a major challenge (Mohamed et al., 2017). A recent study explored production of catechol in *E. coli* from vanillin, a common lignin-derived chemical. The aromatic transporter *CouP* was co-expressed with *LigV* and *LigM* for vanillin degradation and the protocatechuate decarboxylase *AroY* in *E. coli* all under a ADH7 vanillin inducible promoter to regulate inhibition and ultimately reduce toxicity (Wu et al., 2018). While successfully increasing catechol titer by 40%, final titers remained comparatively lower than the titer from *P. putida*. Nonetheless, these studies collectively demonstrate how adaptive evolution paired with product-oriented metabolic engineering can valorize lignocellulosic biomass into useful bioproducts.

To summarize, most approaches to valorize lignocellulosic biomass either integrate production pathways into organisms such as *P. putida* and *R. toruloides* with natural tolerance to aromatic compounds or engineer tolerance toward aromatic compounds in common production chassis like *E. coli*. While both have made significant strides over the last decade, most lignocellulosic bioproduction platforms are not yet commercially viable.

### C1 substrates

Many C1 substrates are generated through anthropogenic waste streams in the form of flue gas (CO_2_, CH_4_, and CO), anaerobic digestion (CH_4_, CO_2_), and as byproducts of the petroleum industry (CH_4_). CO_2_ may then be electrochemically converted into methanol (CH_3_OH) and formate (HCOOH). Collectively, this library of chemicals has the potential to revolutionize value-added production by methylotrophic, formatotrophic, or phototrophic organisms. Methane and methanol are especially appealing due to their high energy-to-carbon ratios compared to glucose such that pairing methylotrophy with glucose consumption could balance production, a framework that has already demonstrated improvements in titers and yields while reducing costs (Liu et al., 2020).

Although at the Frontier of microbial bioproduction, substrate utilization is challenged by the unique physical constraints of C1 chemicals, which demand specialized fermentation reactors, media, and culture conditions. While CO_2_ may be bubbled directly into a reactor, for example, photosynthetic organisms require sufficient illumination that increase production costs dramatically. Likewise, carboxidotrophic bioproduction is hampered by the low solubility of CO in aqueous media requiring specialized reactors with high gas-liquid volumetric transfer coefficients to maximize solubility (Köpke et al., 2011a). Furthermore, the use of CH_4_ or CO in any fermentation strategy raises significant safety concerns at scale and, conversely, formic acid and methanol maintain a high energetic cost of synthesis and microbial toxicity, respectively. Nonetheless, these pathways enable the valorization of industrial waste otherwise emitted into the atmosphere and represent a tremendous opportunity from a TEA/LCA perspective.

As with lignocellulosic biomass, the two predominant strategies of C1 bioproduction are through engineering metabolically tractable chassis like *E. coli* and *S. cerevisiae* for C1 assimilation or through heterologous expression of production pathways in native assimilators. Here, we subdivide microbial C1 metabolism primarily into photoautotrophic, methylotrophic, and formatotrophic carbon assimilation with an emphasis on recent advances and improvements.

#### Carbon dioxide

Photoautotrophic microbes photosynthetically fix carbon dioxide to generate carbohydrates via the Calvin-Bassham-Benson (CBB) cycle. Photosynthetic bioproduction is especially appealing due to the conversion of CO_2_ and sunlight into valuable products. Select species of algae (e.g., *Chlorella* sp., *Duniella* sp., *Nannochloropsis* sp., etc) are attractive due to their ability to quickly accrue biomass that, depending on the strain and culture conditions, is often rich in triglycerides and carbohydrates ideal for low value, high volume catalytic conversion. Their natural accumulation of high energy metabolites is further complemented by their uncanny ability to thrive on wastewater, saline, or freshwater environments. From a synthetic biology perspective, the model alga *Chlamydomonas reinhardtii* has a well characterized metabolic toolkit for genetic modification (Tran and Kaldenhoff, 2020), but the translation of these tools and strategies to other algal biofuel candidates has proven extraordinarily challenging (Mosey et al., 2021). Counterintuitively, the greatest improvement in algal biomass accumulation stemmed from a reduction in the light harvesting antenna size. Large light harvesting complexes, for example, enable growth in low light conditions but may lend to high photon absorption and photochemical quenching to avoid photodamage under high light conditions (Kirst et al., 2014). By limiting the potential burden of photon overabsorption in high light conditions, synthetic biologists circumvented the necessary dissipation of excess photons and improved culture biomass accumulation (Melis, 1999; Polle et al., 2003).

As prokaryotes, cyanobacteria (especially *Synechococcus elongatus*, *Synechocystis* sp.) are comparatively easy to genetically modify and maintain a high theoretical photosynthetic energy conversion efficiency of ∼10% (Lewis and Nocera, 2006). As with algae, engineering photosynthetic efficiency is complicated by evolutionary adaptations to natural light cycling, where organisms must propagate under both indirect and direct sunlight. As a result, cyanobacterial photosynthetic efficiency is typically 1–2%. Following promising improvements in algae, *Synechocystis* was also modified with truncated light harvesting antennae, yielding a 57% increase in productivity in limited to high light conditions (Kirst et al., 2014). More recently, sink engineering has also arisen as a novel strategy for rerouting carbon or electron flux towards specific chemicals. Analogous to antenna size reduction, sink engineering reroutes utilizable photosynthetic energy towards bioproducts. The concept has been demonstrated with sucrose (Ducat et al., 2012), 2,3-butanediol (Oliver et al., 2013), and isoprene (Lindberg et al., 2010) through a hypothesized reduction of accumulating inhibitory intermediates. One work also combined sucrose export and P450 expression, noting an additive benefit that further increased photosynthetic efficiency by improving utilization of the electron transport chain (ETC) (Santos-Merino et al., 2021). As a whole, these works establish that heterologous bioproduction or fine tuning of metabolic pathways in canonical cyanobacteria enable access to excess energy from photosynthesis otherwise dissipated to avoid overreduction of the ETC, accumulation of reactive oxygen species, photodamage, and photoinhibition (Santos-Merino et al., 2021). Emerging insight of cyanobacterial photosynthesis is coupled with a slew of metabolic engineering tools like promoter libraries, CRISPR-cas systems, and genome scale models (Santos-Merino et al., 2019) and, collectively, these works provide encouraging strategies of how conditional rewiring of photosynthetic machinery can spur advancements in cyanobacterial bioproduction.

Many thorough reviews, LCAs, TEAs, and subsequent harmonization reports have investigated varied photoautotrophic biofuel production methodologies in an attempt to better understand limitations barring commerciality (Quinn and Davis, 2015; Tu et al., 2017; Cruce et al., 2021). In general, the scaling of algal platforms has proven economically prohibitive due to high CAPEX of the cultivation methodology (e.g., outdoor raceways, photobioreactors, or cattle tanks) combined with culture instability and relative extraction efficiencies. Overall, the most prohibitive obstacles are slow growth, tolerance to environmental fluctuations, and resistance to invasion by grazers, disease, or other phototrophs. While clever strategies have been developed to address culture instability, including polycultivation of multiple strains to improve culture stability (Narwani et al., 2016) or unnatural phosphite feeding (González-Morales et al., 2020), few have proven immediately viable at scale (Carruthers et al., 2019). Thus while rough approximations of, for example, *S. elongatus* sucrose yield outstrip sugarcane on a per hectare basis (Ducat et al., 2012), the realization of these yields in the context of LCA and TEA demands a more holistic engineering approach that integrates laboratory strain improvements and practical cultivation strategies within the biorefinery framework.

#### Methane and methanol

Methylotrophs are enticing candidates for capturing and valorizing industrial methane waste streams. Common candidates include the bacteria *Methylorubrum extorquens* AM1 (formally *Methylobacterium extorquens* AM1) and *Methylobacillus glycogenes* as well as the prominent yeast *Pichia pastoris* (now *Komagataella phaffi*), which incorporate methane or methanol through a variety of highly regulated processes. All methylotrophic organisms first oxidize methanol to formaldehyde through a cofactor dependent oxidoreductase. In bacteria, formaldehyde may be assimilated into carbon metabolism through either the ribulose monophosphate (RuMP) cycle (Type I) or the serine cycle (Type II). Alternatively, yeasts compartmentalize methanol-derived metabolites and ultimately assimilate formaldehyde in the xylulose monophosphate (XuMP) cycle. *K. phaffi*, for example, natively expresses methanol inducible alcohol oxidase genes *AOX1* and *AOX2* to enable methanol assimilation through peroxisome biogenesis, thereby sequestering toxic formaldehyde and hydrogen peroxide (Peña et al., 2018). Leveraging the tightly regulated inducible *AOX1* promoter has enabled biphasic culturing strategies and heterologous production, usually entailing an initial glycerol growth phase followed by a methanol production phase. Recent developments have led to rapid expansion of the methylotrophic production portfolio, including terpenoids ranging from C5 isoprene to C30 squalene, though with lower comparative titers to traditional cultivation (Carruthers and Lee, 2021).

Recently, interest in synthetic methylotrophs has led to metabolic engineering of *E. coli*, *S. cerevisiae,* and *Cornyebacterium glutamicum*. In bacteria, *de novo* C1 assimilation has been achieved through heterologous expression of a methanol dehydrogenase, which enabled growth upon C13 labeled methanol with limited sugar supplementation and subsequent metabolomic profiling of central carbon metabolism (Tuyishime et al., 2018; Kim et al., 2020). From there, various studies have investigated formaldehyde assimilation via native aldolases into the homoserine cycle, or further oxidation to formate, discussed later (He et al., 2020). On the other hand, a recent study identified that *S. cerevisiae* has native tolerance to methanol as well as low levels of assimilation. Methanol assimilation was enhanced by 44% using ALE (Espinosa et al., 2020). Heterologous expression of C1 assimilation pathways has tremendous potential for reducing the economic and environmental burden that have hitherto limited commercial viability, however many obstacles remain to actualize C1 bioproduction including, as an example, safely scaling methane fermentation systems.

#### Formate

Many organisms are capable of oxidizing formate to CO_2_ via a formate dehydrogenase either as a tolerance mechanism or to supply reducing power. Native formate assimilation is usually accomplished by either the reductive pentose phosphate cycle (CBB cycle), the serine cycle, or the reductive acetyl-CoA pathway with varied efficiencies and bottlenecks. Yet few organisms are naturally capable of surviving solely on formate and formate assimilation is phenotypically isolated to a subset of fastidious microbes (Yishai et al., 2016).

Fortunately, formaldehyde assimilation pathways are necessary for methylotrophy. Elucidation of formate and formaldehyde assimilation has coincided with a revolution in our understanding of metabolic pathways. Combinedly, these advances have led to hypothetical synthetic pathways designed to tune thermodynamic efficiencies and redox balances (Bar-Even, 2016; Yishai et al., 2016). Of those synthetic pathways, the reductive glycine pathway (rGly) has proven a popular *in silico* target due to its relatively few enzymatic steps and favorable ATP/NAD(P)H consumption. Remarkably, a novel natural pathway reducing CO_2_ to formate via the rGly was recently elucidated in an isolate of dissimilatory phosphite-oxidizing microorganisms (Figueroa et al., 2018), specifically *Desulfovibrio desulfuricans* (strain G11) (Sánchez-Andrea et al., 2020), demonstrating the propensity of the rGly to naturally drive central carbon metabolism. Indeed, *de novo* formate assimilation pathways in *E. coli* and *S. cerevisiae* have demonstrated *in vivo* serine and glycine production (Yishai et al., 2018; Gonzalez de la Cruz et al., 2019). One seminal study combined overexpression of the rGly cycle with ALE to yield a strain of *E. coli* capable of growth solely on formate and establishing a novel strategy for reduced feedstock cost in a common metabolic chassis (Kim et al., 2020). The confluence of biological and computational approaches in the elucidation and application of the rGly also has tremendous implications for further pathway optimization. It also presents an opportunity for rewiring or heterologous expression of individual genes from the remaining four CO_2_ fixation pathways (the reductive TCA cycle, the 3-hydroxypropionate cycle, dicarboxylate/4-hydroxybutyrate cycle, and the 3-hydroxypropionate/4-hydroxybutyrate cycle).

Complementary to biological advances, LCAs have shown that electrochemical derivation of formic acid from CO_2_ is far more dependent on grid specific electricity composition compared to other C1 chemicals. As electricity and H_2_ are increasingly generated from a renewable grid, formic acid becomes increasingly viable as a substrate (Sternberg et al., 2017). Integration and tuning of *de novo* formate assimilation pathways represents the Frontier of feedstock engineering and, in the context of advances in carbon sequestration, could enable upcycling of CO_2_ into valuable chemicals.

#### Carbon monoxide

Carbon monoxide is a major constituent of syngas (CO_2_, CH_4_, H_2_, and CO) commonly generated through gasification reactions of biological or fuel carbon or through steam reforming in ammonia synthesis. Historically, the value of syngas lies in its canonical catalytic conversion into valuable hydrocarbons through the Fischer-Tropsch process or into hydrogen via the water-gas shift (Teixeira et al., 2018). Yet, from a microbial perspective, syngas represents a unique feedstock for niche anaerobic. CO metabolism is accomplished anaerobically or aerobically through the expression of a specialized heterometaloenzyme, carbon monoxide dehydrogenase (CODH). Microbial CODHs are classified as either monofunctional or bifunctional. Monofunctional CODHs reversibly oxidize CO to CO_2_ whereas bifunctional CODHs, coupled with an acetyl-CoA synthetase, also catalyze the condensation of CO, a CO_2_ derived methyl group, and a CoA-SH to generate acetyl-CoA (Oelgeschläger and Rother, 2008; Schuchmann and Müller, 2014).

Carboxidotrophs represent a specific class of bacteria capable of sole growth on CO through its oxidation to CO_2_ and subsequent incorporation into central carbon metabolism via the reductive acetyl-CoA pathway. Reducing equivalents from CO oxidation then drive respiratory functions with further capacity to generate hydrogen, acetate, methane, and so forth (Oelgeschläger and Rother, 2008; Schuchmann and Müller, 2014). As a result, this unique metabolic class is promising in its capacity to further valorize syngas. Microbial growth on CO_2_, CO, and H_2_ principally using *Clostridium* sp. Has demonstrated production of isoprene, 2,3-butanediol, ethanol, succinic acid among other products (Köpke et al., 2011b; Liew et al., 2016). Commercial interest by LanzaTech (Köpke et al., 2011a; Heijstra et al., 2017) has spurred strain development and pilot coupling of microbial systems with industrial waste. Although much of the information is proprietary, a recent LCA of the LanzaTech process asserted that their ethanol production platform could generate ethanol from syngas at 60% reduced GHG emissions compared to conventional processes, a figure that matches the EPA standards set for cellulosic biomass (Handler et al., 2016). Importantly, the diversity of microbial C1 metabolism presents a fascinating opportunity for valorizing syngas beyond conventional conversion approaches as we describe in the following section.

### Consortial approaches

Engineered microbial communities divide a metabolic pathway between constituent organisms to increase overall pathway efficiency. Consortial approaches may integrate microbial and chemical syntheses into a single, unified platform that parallels agricultural or industrial waste stream effluxes (Roell et al., 2019). Emblematic of natural ecological processes like lignolytic detritus degradation, synthetic biologists can co-culture microbes to access recalcitrant substrates. Pairing *Trichoderma reesei*, a filamentous yeast renowned for secreting cellulolytic enzymes, with *E. coli,* for example, enabled access to sugar monomers and ultimately to 1.88 g/L isobutanol production (Minty et al., 2013).

More recently, researchers have engineered consortia with C2 metabolite cross feeding (Kim Y. et al., 2021; Ma et al., 2021). Ethanol and acetate are typically generated via overflow metabolism, which is characterized by incomplete oxidation of sugars in high growth rate production organisms, or by acetogenic microbes under anaerobic conditions. However, assimilation of C2 substrates is challenging due to imbalances in ATP, NADPH, and acetyl-CoA generation. Acetate, for example, may be directly converted into acetyl-CoA, but ATP and NADPH generation typically require the addition of sugars (Park et al., 2019). A specific study expressed acetaldehyde and alcohol dehydrogenase enzymes *ada* and *adha*1 in *E. coli* for direct conversion of ethanol to acetyl-CoA, demonstrating low titer production of PHB and prenol in rich medium (Liang et al., 2021). Co-feeding of acetate and gluconate in *Y. lipolytica* has also been reported for lipid production in which the low NADPH barrier is overcome by limited sugar addition, presenting its possible candidacy in a microbial consortia (Park et al., 2019).

Pairing specialized microbes has emerged as a strategy to reduce nutrient input costs as in the case of photoautotrophic and diazotrophic bacteria, which natively fix carbon dioxide and nitrogen gas, respectively. Fixation and excretion of sucrose by *S. elongatus* demonstrated co-culture production of low titer PHBs from an engineered *P. putida* (Löwe et al., 2017) and native *Halomonas boliviensis* (Weiss et al., 2017). Similarly, biologically fixed and subsequently secreted nitrogen from *Azotobacter vinelandii* promoted syntrophic growth with green algae, thereby reducing Haber-Bosch derived nitrogen costs (Villa et al., 2014). Following these examples, microbial consortia can extend to more complex systems like engineered mycorrhizal communities that improve agricultural productivity while reducing input costs (Garrido-Oter et al., 2018; Wurtzel et al., 2019).

Valorization of waste streams is an obvious yet challenging opportunity for reducing production cost and environmental burden. An important caveat is that the success of these microbial platforms appears contingent on coupling bioproduction with traditional energy waste streams like flue gas. As energy systems continue to decarbonize, these platforms may adapt through cross-platform consortia that pair electrochemical processes like carbon capture and storage (CSS) and CO_2_ hydrogenation with microbial fermentation (Bui et al., 2018). Of special interest are combinatorial approaches using CSS technologies to convert sequestered carbon into bioproducts (Faber et al., 2021) in “bioenergy CSS” platforms (Hanssen et al., 2020). Many proof of concept studies have already been established, including electrochemical conversion of CO_2_ to formic acid for bioproduction in *C. necator* (Li et al., 2012).

Assessments of multifaceted systems are inherently challenging due to necessary approximations. For example, fluctuations in temporal microbial composition of mycorrhizal consortia must be reduced to generalized terms (e.g., productivity, yield, elemental composition). The challenge is therefore in summarizing complex processes without compromising on data resolution to elucidate important bottlenecks with some certainty, a challenge that may require more specialized tools within the LCA-TEA community.

## Biofuels, biopolymers, and precursors

The substitution of petrochemicals with biobased sources is a major opportunity for reducing global GHG emissions (Zheng and Suh, 2019). In 2004, the United States Department of Energy published a report of fifteen chemical targets for biorefineries (Werpy and Petersen, 2004), which was further updated in 2010 based on the current state of technology. The chemicals are categorized by nine technological criteria that range from possible co-products in a scalable biorefinery to conversion and TRY (Bozell and Petersen, 2010). More recently, efforts have shifted to the concept of the BioFoundry that can rapidly generate an array of products from beachhead molecules, which include metabolic precursors like pyruvate, acetyl-CoA, malonyl-CoA, and the like (Hillson et al., 2019; Benavides et al., 2021). Importantly, these projects embody the use of LCA/TEA to streamline and productionize synthetic biology, while explicitly considering non-model organisms and atypical carbon substrates (Benavides et al., 2021).

It is notoriously difficult to compare bioproduction LCA/TEAs due to critical differences in assumptions, parameters, and process-level design decisions. More exhaustive meta-analysis and critical reviews of assessment strategies tend to highlight limited LCI data or sparsity of available assessments in general. Parsing variability between studies to determine process viability or sustainability is extremely challenging and often requires product specific reviews with a case-by-case analysis of LCA/TEA (Ögmundarson et al., 2020).

While more general reviews of bio-feedstocks are available (Cywar et al., 2021; Keasling et al., 2021), here we highlight a limited subset of biochemical precursors leveraged for bulk microbial production (lactic acid, succinic acid, adipic acid, 3-hydroxypropionic acid, and l-lysine), biopolymers (polyhydroxyalkanoates), and isoprenoid biofuels (bisabolene, limonene, and isoprenol) with specific attention to recent improvements in TRY, growth on non-glucose substrates, metabolically proximal co-products, and studies that include LCA/TEA. Rather than focusing on actual assessments, we consider how the metabolic engineering of strains for improved characteristics could translate into increased economic and environmental performance at scale.

## Biochemical precursors

### Lactic acid

Many industrial schemes have explored lactic acid production due in part to the natural abundance of lactic acid accumulating bacteria (e.g., *Lactobacillus* sp., *Lactococcus* sp., *Lacticaseibacillus* sp.) that generally outcompete pathway expression in common bioproduction chassis. Lactic acid is typically produced by a reduction of pyruvate under anaerobic conditions and can be readily condensed to polylactic acid (PLA) either through direct condensation or a ring-opening reaction involving the lactide intermediate (Vink et al., 2003). PLA is an attractive biopolymer not only due to its comparable thermal and mechanical properties to polystyrene and polyethylene terephthalate, but its high biodegradability (Zheng and Suh, 2019).

An LCA predating ISO 14044 was published to measure the relative GHG and energetics of the process outlined by Cargill Dow’s NatureWorks™ PLA in 2003 (Vink et al., 2003) with estimated GHG emissions of 1.6 kg CO_2_eq/kg PLA and with a required 54 MJ/kg, astonishing figures that outstripped their displaced plastic counterparts. Notably, these metrics stem from the cradle-to-gate system boundary, few process details, and lack of sensitivity data. Despite these clear deficiencies, the primordial LCA established feedstocks as a major opportunity for improving sustainability metrics and advocated that transitioning from pure sugars to corn stover could provide a 10-fold reduction in process energy demand. Many publications have since varied production strategies on glycerol and lignocellulosic biomass with a veritable portfolio of production organisms (Morales et al., 2015; Adom and Dunn, 2017; Li et al., 2021). In particular, a recent work demonstrated production of 0.6 g/L of lactate from methane by *Methylomicrobium buryatense* with a TEA yielding optimistic values of 5.83–2.17 $/kg MSP, approaching those of lignocellulosic-based production (Garg et al., 2018; Fei et al., 2020).

### Succinic acid

As with lactic acid, succinic acid (SA) is shortlisted as a top bulk biochemical precursor for use in generation of polybutylene succinate, polyester polyols, polyurethanes, 1,4-butanediol, and adipic acid among other chemicals (Jansen and van Gulik, 2014). Bio-succinic acid production has steadily increased over the last decade and culminated in a number of burgeoning commercial platforms via heterologous production in *E. coli*, *Actinobacillus succinogenes,* and *C. glutamicum* (Nghiem et al., 2017; Dickson et al., 2021). Optimization strategies typically involve channeling metabolic flux towards succinate by elimination of alternative anaerobic byproduct pathways, often down-regulating or completely removing native *ldh*, *acka*, *pta*, and *pfl*, which encode for a lactate dehydrogenase, acetate kinase, phosphate acetyltransferase, and formate acetyltransferase, respectively. Candidate strains have historically achieved titers close to or greater than 100 g/L and recent SA LCAs have tended to deem competitive or comparable to petrochemical pathways (Ögmundarson et al., 2020).

One study introduced the *M. extorquens* gene *fhd*2 for formic acid assimilation into a strain of *Mannheimia succiniciproducens* with significant modifications to mixed acid fermentation pathways (Ahn et al., 2017). Although supplementing formic acid and mixed sugars at a 1:5 ratio, the authors ultimately demonstrated 76.1 g/L SA production (4.08 g/L/h and 1.28 M yield) with C13 analysis (Ahn et al., 2017), approaching conventional production on glucose (Ögmundarson et al., 2020). Furthermore, TEAs of lignocellulosic biomass derived biofuel production have also highlighted SA as an exemplary value-added co-product for improved process valorization, which may prove pivotal for achieving favorable economics in other production pathways (Biddy et al., 2016).

### Adipic acid

Approximately three million tons of adipic acid are generated annually, mainly to produce nylon (Kruyer and Peralta-Yahya, 2017). Adipic acid is generated through chemical synthesis using nitric acid and cyclohexane, generating nitric oxide as an extremely potent GHG byproduct in unabated systems (Kruyer and Peralta-Yahya, 2017). A plethora of biosynthetic pathways have been designed for adipic acid production and are generally divisible into production of muconic acid and glutaric acid precursors or direct production of adipic acid itself (Kruyer and Peralta-Yahya, 2017; Skoog et al., 2018). Engineering approaches for muconic acid have successfully utilized an extended shikimate pathway in which catechol is generated from chorismate either via a variety of intermediates including salicylic acid 4-hydroxybenzoic acid, 2,3-dihydroxybenzoic acid, or protocatechuic acid (Kruyer and Peralta-Yahya, 2017). Catechol is then converted to muconic acid via a heterologous catechol 1,2-dioxygenase with titers in *E. coli* reaching 36.8 g/L *cis,cis*-muconic acid (Niu et al., 2002). Other works have investigated direct adipic acid production via the reverse β-oxidation pathway, ω-oxidation pathway, and reverse adipate degradation pathway. By heterologously expressing a combination of five key genes from *Thermobifida fusca* and a CRISPR-mediated deletion of *ldhA*, *sucB*, and *atoB*, the latter approach ultimately generated 68.1 g/L adipic acid (0.381 g-adipic acid/g-glucose) in super rich medium (Zhao et al., 2018).

A recent TEA noted that, assuming theoretical yields of adipic acid and excellent catalyst properties, a fully biologically derived adipic acid route could achieve 1.36 $/kg, well below the MSP of 1.60 $/kg (Gunukula and Anex, 2017), but obtaining such biological yields is extraordinarily challenging. Alternatively, an estimated 41.79% of total adipic acid expenditures stem from growth on sugar feedstocks (Johnson et al., 2019). Lignin-based production, albeit at very high efficiencies, could achieve prices as low as 0.88 $/kg adipic acid (Rios et al., 2021). Furthermore, generation of adipic acid from lignin could reduce an estimated 62%–78% emissions compared to chemical synthesis (Corona et al., 2018). *P. putida* has arisen as a promising candidate in addressing feedstock burden due to its genetic tractability and native resistance to lignin aromatic toxicity. Following this predisposition, an engineered strain of *P. putida* demonstrated 13.5 g/L titer from M9 minimal medium fed with a 2:1 glucose to *p*-coumaric acid ratio (Vardon et al., 2015). More recently, an investigation demonstrated low titer muconic acid production in a modified *P. putida* grown solely on variably sourced lignocellulosic hydrolysate without exogenous sugar supplementation (Sonoki et al., 2018). Crucially, these advances move biologically derived adipic acid towards higher TRL and potential viability at scale.

### 3-Hydroxypropionic acid

Generation of 3-hydroxypropionic acid (3-HP) is achieved firstly through the dehydration of glycerol via a B12-dependent dehydratase and reduction by an alcohol dehydrogenase (Nakamura and Whited, 2003). The major derivatives of 3-HP include other bulk biochemicals like acrylic acid, 1,3-propanediol (1,3-PDO) and 3-hydroproprionaldehyde. For many anaerobic organisms, production of 3-HP from glycerol serves as an electron sink to enable NAD + regeneration (Nakamura and Whited, 2003). 1,3-propanediol (1,3-PDO) is of special interest as a product of 3-HP reactions due to its potential as a biopolymer precursor, the proprietary generation of which has been completed biologically in *E. coli* by DuPont for several decades on glucose (Nakamura and Whited, 2003; Bozell and Petersen, 2010). More recently, *C. glutamicum* derived 1,3-PDO from glucose has demonstrated a final titer of 110.4 g/L, a yield of 0.42 (g-1,3-PDO/g-glucose), and a productivity of 2.30 g/L/h in fed-batch fermentation (Li et al., 2022). By further integrating xylose metabolism for simultaneous uptake of glucose and xylose, 1,3-PDO production from mixed sugar approached those metrics from pure glucose (98.2 g/L vs. 110.4 g/L titer, 0.38 (g-3-HP/g-mixed substrate) vs. 0.42 (g-3-HP/g-glucose yield)) (Li et al., 2022).

In an utterly different strategy, 3-HP production has been demonstrated in the Type II methanotroph *Methylosinus trichosporium* OB3b. Type II methanotrophs maintain high acetyl-CoA flux, which is a particularly useful trait in deriving valuable chemicals from C1 feedstocks (Nguyen et al., 2021). By engineering the malonyl-CoA pathway, methane fed cultures in nitrate mineral salt medium achieved 60.59 mg/L 3-HP (Nguyen et al., 2020). An optimized titer of 69.8 mg/L 3-HP has also been demonstrated in the methylotroph *M. extorquens* on a supplemented minimal medium (Yang et al., 2017). While low, such works demonstrate feedstock ingenuity and the potential for less common organisms to serve as metabolic chassis.

Finally, an assessment of 3-HP derived acrylic acid in BioSTEAM for lignocellulosic substrates noted a baseline MSP of 1.83 $/kg, assuming a titer of 54.8 g/L, productivity of 0.76 g/L/hr, and theoretical yield of 49% in *C. glutamicum* on lignocellulosic glucose and xylose (Bhagwat et al., 2021). Although marginally higher than current market prices, Bhagwat et al. establish critical advances necessary to achieve market competitiveness. As a result, the recent improvements in mixed substrate *C. glutamicum* yields are especially encouraging.

### l-lysine

l-Lysine is an essential amino acid and critical precursor to several industrially relevant chemicals including glutaric acid, diamines, and 5-aminolevulinic acid (5-AVA), which are of special interest in their polymerization to polyamides, namely Nylon-6 and Nylon-510, which are almost ubiquitously derived from petrochemicals. *E. coli* has commonly been employed as a chassis for lysine derivatives with significant lysine supplementation. The heterologous expression of *P. putida* genes *davB* and *davA* for a lysine monooxygenase and delta-aminovaleramidase, respectively, in lysine supplemented medium led to 3.6 g/L 5-AVA production (Park et al., 2013). Further addition of the *P. putida* 5-AVA aminotransferase, glutarate semialdehyde dehydrogenase, and supplementation with alpha-ketoglutarate led to 1.7 g/L glutarate production (Park et al., 2013). The diamine cadaverine has been generated in *E. coli* through overexpression of the lysine production pathway, diamine degradation knockouts, and heterologous expression of the lysine decarboxylase *cadA* for conversion of lysine to cadaverine (Qian et al., 2011). Ultimately, Qian et al. demonstrated 9.62 g/L cadaverine production at a rate of 0.32 g/L/h and 0.12 (g-lysine/g-glucose) yield on minimal medium without lysine supplementation (Qian et al., 2011).

More recent works have explored polyamide precursor production in *C. glutamicum*, which naturally accumulates l-lysine due to a lack of native degradation enzymes (Kalinowski et al., 2003). Iterative optimization of *C. glutamicum* has included tuning the pentose phosphate pathway for improved NADPH cofactor production and a systematic overexpression of lysine biosynthesis genes, namely *lysA*, *dapB*, *lysC*, and *ddh* coupled with reduced expression of threonine dehydrogenase and specific TCA modification (Becker et al., 2011). Collectively, these modifications resulted in fed-batch production of 0.55 g-lysine/g-glucose and a final titer of 120 g/L lysine at a production rate of 4 g/L. While *C. glutamicum* appears an obvious chassis for lysine derivatives, initial generation has been hampered by co-production of non-target derivatives, like N-acetylcadaverine in the case of cadaverine production (Kind et al., 2010). Nonetheless, *C. glutamicum* maintains significant resistance to 5-AVA and glutarate toxicity and, unlike the diamines, demonstrated tunable product selectivity (Rohles et al., 2018).

Lysine derivatization poses a unique dilemma between organisms, specifically high precursor production, production on inexpensive substrates, and optimization of derivative pathways. More recently, *C. glutamicum* showed high titer conversion of lysine to glutaric acid (105.3 g/L), which is recognized as an important chemical precursor to polyamides and polyurethanes (Bozell and Petersen, 2010; Han et al., 2020). Nylon precursor production has also been demonstrated on nonsugar substrates like methanol and CO_2_ though at markedly lower concentrations of 6.5 g/L cadaverine in *Bacillus methanolicus* (Naerdal et al., 2015) and 1.74 mM lysine in *Synechococcus* sp. (Korosh et al., 2017), respectively.

## Isoprenoid biofuels

Biofuels comprise a broad category of drop-in chemical compounds that may serve as fuel additives to improve fuel characteristics (e.g., octane and cetane numbers, oxygen sensitivity, engine performance) or supplement conventional diesel/gasoline entirely. Advanced biofuels are produced from inedible carbon substrates and are especially attractive due to their propensity to displace conventional fossil fuels while valorizing the waste streams described previously. Advanced biofuels derived from metabolic routes for isoprenoids, fatty acids, branched amino acids, and ketones, have arisen as important candidates in the energy market (Keasling et al., 2021).

Isoprenoids are naturally derived from C5 precursors (isopentenyl diphosphate (IPP) and dimethylallyl diphosphate (DMAPP)), which are in turn generated through either the mevalonate (MVA) or the methylerythritol 4-phosphate (MEP) pathways. While these pathways maintain different efficiencies, cofactors, and initial precursors, both have been candidates of extensive optimization to produce pharmaceuticals, fragrances, solvents, and biofuels. While hundreds of thousands of terpenes exist, biofuel candidates are generally limited to C5-C15 chemicals, especially those leveraged for bulk microbial production. Of special interest are the expansion of isoprenoid production platforms to non-model microbial chassis including *R. toruloides*, *Y. lipolytica*, as well as production on C1 carbon substrates (Carruthers and Lee, 2021). Major isoprenoid-derived biofuel candidates may be classified by chain length and include the hemiterpenes (C5) isoprenol, prenol, and isoprene, the monoterpenes (C10) limonene and 1,8-cineole, and the sesquiterpenes (C15) bisabolene and farnesene, among others.

From an energetic perspective, microbial biofuel production is a biotransformation of a feedstock—a critical reason why photoautotrophic cyanobacteria and algae have historically attracted significant research attention. On the other hand, methylotrophs or lignolytic organisms must also compete thermodynamically with simply combusting lignocellulose or methane, respectively. As with any energy transformation, the efficiency or energy return on energy invested (EROI) of these bioconversions may dictate their viability (Fei et al., 2014). While most microbial biofuel platforms continue to use pure C5 and C6 sugar feedstocks, they remain instrumental to actualizing advanced biofuel production. Here, we focus on isoprenoid biofuels that have demonstrated high titer and are approaching commercial viability.

### Bisabolene

While isoprenoid derived biofuels have attracted significant attention as fuel enhancing additives, their titers and scalability of current technologies remain variable. Bisabolene, for example, demonstrated yields of approximately 1 g/L in *S. cerevisiae* and *E. coli*, by careful MVA pathway balancing and quorum sensing mediated pathway expression, respectively (Peralta-Yahya et al., 2011; Kim et al., 2017). Production has also expanded into *R. toruloides*, an oleaginous yeast with attractive natural characteristics for production on unfiltered sorghum hydrolysate (Sundstrom et al., 2018). Optimization of this microbe with multiple genomic copies of the MVA pathway resulted in 2.6 g/L bisabolene in a 2-L fermentation reaction, accounting for approximately 10% theoretical yield (Kirby et al., 2021).

### Limonene

Production of limonene in *E. coli* was demonstrated by Alonso-Gutierrez et al. via fine tuning of MVA pathway genes on a plasmid also harboring a limonene synthase from *Mentha spicata* and a truncated geranyl diphosphate synthase from *Abies grandis* (JBEI-6410). Pathway expression in *E. coli* DH1 ultimately achieved a titer of approximately 435 mg/L limonene on 1% glucose (compared to 0.32 g-limonene/g-glucose theoretical maximum) (Alonso-Gutierrez et al., 2013) with a similar titer recently achieved in *R. toruloides* (Liu S. et al., 2021). Titer was further improved to 3.6 g/L by tuning culture conditions with mixed glucose and glycerol feeding in *E. coli* BL21 (DE3) (Rolf et al., 2020). The dramatic improvement was hypothesized to derive from combined flux through both the endogenous MEP and heterologous MVA pathways (Rolf et al., 2020). Lastly, a recent work explored expression in *S. cerevisiae* by exploiting metabolite sequestration to peroxisomes, thereby partitioning product toxicity while maintaining proximal generation of acetyl-CoA (Dusséaux et al., 2020). The approach ultimately achieved a titer of 2.6 g/L under fed-batch conditions in synthetic medium (Dusséaux et al., 2020).

These recent improvements have made remarkable strides towards economic viability, though TEAs still estimate the MSP of limonene to be between 20 $/kg and ∼7 $/kg if yields are improved to 45% (0.144 g-limonene/g-glucose) (Sun et al., 2020) or 30% (0.096 g-limonene/g-glucose) if coupled with significant feed and culture optimizations (Wu and Maravelias, 2018). Both figures are far higher than current methodologies, though highlight the burden of pure sugar substrate on overall cost.

### Isoprenol

The C5 alcohols isoprenol and prenol have enormous potential as biofuel additives and precursors. Prenol has demonstrated a unique blendstock characteristic called hyperboosting in which its addition increases the research octane number (RON) of the blendstock above the RON of the individual components (Monroe et al., 2019). On the other hand, isoprenol can also serve as a precursor to 1,4-dimethylcyclooctane (DMCO), a drop-in jet fuel (Baral et al., 2021). From a metabolic perspective, isoprenol is generated by a simple sequential dephosphorylation of IPP. However, IPP accumulation is inhibitory such that high titer isoprenol bioproduction has proven challenging. Recently, heterologous expression and subsequent mutagenesis of the *S. cerevisiae* mevalonate diphosphate decarboxylase enzyme was demonstrated to avoiding intracellular accumulation of IPP, a known toxic intermediate hypothesized to limit overall isoprenol efficiency (Kang et al., 2017; George et al., 2018). This “IPP-bypass” enhanced isoprenol titer by 2.4-fold to 1.1 g/L (Kang et al., 2017). Further incorporation of an optimized upstream MVA pathway into *E. coli* DH1 also harboring acetate pathway knockouts (Δ*ackA*, Δ*pta*, and Δ*poxB*) resulted in 10.38 g/L titer in 2-L fermenters on minimal medium (0.105 g-isoprenol/g-glucose and a productivity of 0.157 g/L/hr) (Kang et al., 2019). This titer is especially encouraging for production of DMCO, with baseline unoptimized metrics of 9.0 $/L-Jet-A-eq and 61.4 g CO_2_eq/MJ (Baral et al., 2021). Another work demonstrated isoprenol production in yeast by knocking out an endogenous kinase and overexpressing a heterologous phosphatase to yield 380 mg/L isoprenol (Kim J. et al., 2021).

## Polyhydroxyalkanoates

Polyhydroxyalkanoates (PHAs) are a large class of natural polyesters including short chain length (scl) monomers poly (3-hydroxybutyrate) (PHB), poly (3-hydroxyvalerate) (PHV), and their co-polymer PHBV with poor thermal and mechanical properties (Li et al., 2016). PHAs are an amalgam of fatty acids, with C5 and C4 acids generating the scl-PHAs of PHV and PHB, respectively. The fatty acids themselves may be generated through varied metabolic pathways including fatty acid synthesis and β-oxidation, which may also generate medium and long chain length PHAs (Mezzina et al., 2021). A nominal production strategy for PHB is a three-step process initiating with a Claisen condensation of two acetyl-CoA molecules to form acetoacetyl-CoA, reduction to 3-hydroxybutyryl-CoA, and polymerization to PHB via phaA, phaB and phaC, respectively. PHV may proceed via a similar pathway, though with a propionyl-CoA precursor. PHAs are commonly produced by bacteria as a carbon storage mechanism under nutrient stress, though exhibit varying degrees of biodegradability and elasticity due to differences in monomeric composition and chain length. While these characteristics limit the use of PHAs for thermomechanical applications, PHAs have attracted attention as single-use bioplastics for decades.

Accordant with most of the bioproducts described in this review, the commercialization of PHAs has been hampered by feedstock costs, low conversion, and bioprocessing demands amounting to approximately 4–6 $/kg, values far higher than comparable petrochemical products like polyethylene (Chen et al., 2020; Tan et al., 2021). The vast majority of PHA production strategies have been conducted on glucose and are typically limited to *E. coli* and *C. necator*, both of which have achieved titers well over 100 g/L (Zheng et al., 2020; Tan et al., 2021). Metabolic engineering of atypical production organisms, namely the halotolerant *Halomonas* sp., *P. putida*, and *M. extorquens*, has arisen as a promising opportunity for reducing culturing costs and enabling consumption of inexpensive feedstocks. As previously noted, *P. putida* has gained traction as a metabolic chassis due to its metabolism of mixed aromatics and potential for valorizing lignin (Mezzina et al., 2021). Feeding alkaline pretreated liquor, a medium rich in lignin monomers, to *P. putida* demonstrated 34% and 39% conversion of *p*-coumaric acid and ferulic acid to mcl-PHAs, respectively (Linger et al., 2014). Overall titer was then improved to ∼1 g/L mcl-PHAs on pretreated corn stover with 77.6% yield from lignin in the liquid stream (Liu et al., 2017). Using a feedstock of waste cooking oil and a carboxylic acid transport gene pathway knockout (*actA*) resulted in a titer of 1.91 g/L mcl-PHAs (Borrero-de Acuña et al., 2018). Likewise, *M. extorquens* AM1 demonstrated PHA accumulation on methanol, ultimately achieving 43.6% PHAs by weight with 96.6 mol% PHV through overexpression of *phaAB* though at low overall titers (Orita et al., 2014). Although titers in both strains are well below economic viability, they provide low TRL opportunities for improvement and, ideally, significant overall cost reduction.

## Perspectives and conclusion

In this review we have elaborated on the challenges associated with *ex ante* life cycle and technoeconomic analysis of emergent, low TRL biotechnologies while highlighting significant synthetic biology advances that have elevated the sustainable and economic viability of certain bioproduction platforms. Assessments of microbial bioproduction are often surmised by a core set of parameters - feedstock burden as well as fermentation titer, rate, and yield. Advances in metabolic flux analyses, -omics studies, and genome scale modeling have also facilitated construction of computationally informed theoretical or stoichiometric approaches to guide and inform laboratory research. While scaling of the current state of technology still often lends to platform infeasibility, the associated sensitivity analyses are vital for guiding future metabolic engineering and have realized astonishing improvements in many bioproduction pathways.

Nonetheless, the plurality of bioproduction assessments of emergent technologies poses a challenge to comparative analyses due to the sheer range of inputs, strategies, and overall frameworks, which are especially true for LCAs (Cruce et al., 2021). Assessments usually require simplification of the system. The paucity of collaborative studies by experts in the fields of biology and industrial ecology can lend to complications in design accuracy, interpretation, and poor policy decision making (DeCicco et al., 2016). Extrapolating lessons learned from algal biofuels, namely encouraging multidisciplinary collaboration and early LCA-TEA integration to better understand trade-offs, input sensitivity, and viability at scale is necessary for spurring research investment into promising platforms (Mahmud et al., 2021).

Some critics assert that a simple translation of the LCA framework to synthetic biology is fundamentally flawed due to the incompatible extrapolation of materials accounting to biological systems, blurring of ecology and industry, and the intersection of engineered organisms with the natural environment (Seager et al., 2017). And indeed, the recapitulation of the LCA framework for biological systems demands integration not only of industrial ecology and biology, but of physical and social sciences to better inform the implications and applications of synthetic biology, which will hopefully guide governmental policy (Trump et al., 2019). Fortunately, these factors may readily be addressed through careful determination of impact factors. Many LCAs already include esoteric factors like biodiversity loss and human health but have only recently ventured into other critical socio-political factors like environmental justice and a social cost of carbon. Incorporation of a social cost of carbon in assessments will help valorize emissions reduction, facilitating translation between LCA and TEA metrics.

Assessments of bioproduction platforms may also be ameliorated by a combination of transparency and sensitivity to temper theoretical and demonstrated production in the context of environmental risk (e.g., biosecurity, horizontal gene transfer, escape mutants, etc.). The increased uncertainty of bioproduction is a characteristic of biological systems themselves. For example, scaled *E. coli* bioproduction is commonly avoided due to concerns with strain stability, product toxicity, limited feedstock pool, poor production of proteins, and metabolite feedback inhibition (Neubauer et al., 1995; Calero and Nikel, 2019). While the uncertainty is not to be understated, the quantification of economic and environmental tradeoffs is nonetheless critical for benchmarking processes and guiding policies. Effective integration of synthetic biology with environmental and economic assessments is critical for actualizing industrial biotechnology and decarbonizing bulk chemical production.

## Author disclaimer

The views and opinions of the authors expressed herein do not necessarily state or reflect those of the United States Government or any agency thereof. Neither the United States Government nor any agency thereof, nor any of their employees, makes any warranty, expressed or implied, or assumes any legal liability or responsibility for the accuracy, completeness, or usefulness of any information, apparatus, product, or process disclosed, or represents that its use would not infringe privately owned rights.
